# Valproate Use During Spermatogenesis and Risk to Offspring

**DOI:** 10.1001/jamanetworkopen.2024.14709

**Published:** 2024-06-04

**Authors:** Jakob Christensen, Betina B. Trabjerg, Julie Werenberg Dreier

**Affiliations:** 1 Department of Clinical Medicine, Aarhus University, Aarhus, Denmark; 2 Department of Neurology, Aarhus University Hospital, Aarhus, Denmark; 3 National Centre for Register-based Research, School of Business and Social Sciences, Aarhus University, Aarhus, Denmark; 4 Centre for Integrated Register-based Research, Aarhus University, Aarhus, Denmark

## Abstract

**Importance:**

Concerns exist about teratogenic and long-term neurodevelopmental outcomes of paternal use of valproate during spermatogenesis.

**Objective:**

To evaluate the association between paternal use of valproate during spermatogenesis and offspring risk of congenital malformations and neurodevelopmental disorders.

**Design, Setting, and Participants:**

This nationwide cohort study included 1 235 353 singletons born in Denmark between January 1, 1997, and December 31, 2017, identified in the Medical Birth Register; 1336 children had fathers who had filled prescriptions for valproate during spermatogenesis. Congenital malformations were identified in the first year of life and neurodevelopmental disorders were identified from 1 year of age until December 31, 2018. Statistical analysis was performed March 2024.

**Exposures:**

Paternal valproate exposure was defined as fathers who filled 1 or more prescriptions for valproate immediately before or during the time of spermatogenesis (ie, 3 months prior to conception).

**Main Outcomes and Measures:**

Children with major congenital malformations in the first year of life and with neurodevelopmental disorders before death or end of follow-up were identified in Danish health registers. Log-binomial regression was used to estimate adjusted relative risks (ARRs) of congenital malformations, and Cox proportional hazards regression was used to estimate adjusted hazards ratios (AHRs) of neurodevelopmental disorders, adjusted for relevant confounders.

**Results:**

Among 1 235 353 live births (634 415 boys [51.4%] and 600 938 girls [48.6%]), 1336 children (0.1%) had fathers who filled prescriptions for valproate during spermatogenesis. The median follow-up was 10.1 years (IQR, 5.1-14.8 years) for valproate-exposed children and 10.3 years (IQR, 5.2-15.6 years) for valproate-unexposed children. A total of 43 903 children (3.6%) received a diagnosis of major congenital malformations in the first year of life, and 51 633 children (4.2%) received a diagnosis of neurodevelopmental disorders during follow-up. When comparing the risk among valproate-exposed children with that among unexposed children, the ARR of major congenital malformations was 0.89 (95% CI, 0.67-1.18), the AHR of neurodevelopmental disorders was 1.10 (95% CI, 0.88-1.37), and the AHR of autism spectrum disorder was 0.92 (95% CI, 0.65-1.30). In analyses addressing the robustness of the findings (ie, dose-response analyses, sibling analyses, analyses restricted to children of fathers with epilepsy, analyses that used children with paternal lamotrigine exposure as active comparator, and analyses that used children with paternal exposure to valproate only before spermatogenesis as a negative control exposure), there still was no increased risk of any of the included end points.

**Conclusions and Relevance:**

In all analyses based on this large Danish cohort study, results suggest that exposure to valproate during spermatogenesis was not associated with offspring risk of congenital malformations or neurodevelopmental disorders, including autism spectrum disorder.

## Key Points

**Question:**

Is paternal use of valproate during spermatogenesis associated with risk of congenital malformations and neurodevelopmental disorders among offspring?

**Findings:**

In this nationwide cohort study in Denmark comprising 1 235 353 children, including 1336 children born to fathers who filled prescriptions for valproate during spermatogenesis, no association was found between paternal valproate use and risk of major congenital malformations or neurodevelopmental disorders, including autism spectrum disorder.

**Meaning:**

This study suggests that paternal prescription of valproate during spermatogenesis was not associated with risk of major congenital malformations and neurodevelopmental disorders, including autism spectrum disorder, among offspring.

## Teaser

This cohort study of more than 1 million children in Denmark evaluates the association between paternal use of valproate during spermatogenesis and offspring risk of congenital malformations and neurodevelopmental disorders, including autism spectrum disorder.

## Introduction

Epilepsy is one of the most prevalent neurologic disorders among males of fertile age,^1,2,3^ and the antiseizure medication (ASM) valproate is commonly used for epilepsy in males of fertile age.^4,5^ The teratogenic potential of valproate is widely recognized, and use by mothers in pregnancy has been associated with an increased risk of congenital malformations^6,7^ and neurodevelopmental disorders, including, and perhaps most notably, autism among the offspring.^8,9^ Despite the well-known risks associated with maternal exposure to valproate in pregnancy, the risk of congenital malformations and neurodevelopmental disorders associated with paternal exposure in relation to conception is still uncertain. However, recently, the UK Medicines and Healthcare Products Regulatory Agency issued a warning against the use of valproate among males younger than 55 years due to concerns related to male fertility and risk of neurodevelopmental disorders among the offspring.^10^ In addition, the European Medicines Agency’s Pharmacovigilance Risk Assessment Committee (PRAC)^11,12,13^ reported findings from a study based on register data from Denmark, Norway, and Sweden that found paternal valproate exposure to be associated with increased risk of neurodevelopmental disorders (adjusted hazard ratio [AHR], 1.50 [95% CI, 1.09-2.07]) but not congenital malformations (crude odds ratio, 0.81 [95% CI, 0.48-1.36]) among their offspring. These findings and warnings naturally raise concern relating to the use of valproate among fertile men, but association studies of drug effects can be subject to confounding by indication, when the clinical indication for using a drug is independently associated with the study outcomes—that is, if paternal epilepsy is associated with an increased risk of congenital malformations or neurodevelopmental disorders in their children. We therefore specifically applied various approaches known to mitigate confounding by indication in analyses of the association of paternal exposure to valproate in relation to spermatogenesis and risk of major congenital malformations and neurodevelopmental disorders, including autism.

## Methods

### Study Design and Study Population

We conducted a nationwide, population-based cohort study of all singletons born alive in Denmark between January 1, 1997, and December 31, 2017, identified in the Medical Birth Register.^14^ We excluded children with unknown or unlikely values of gestational age (≥315 days or ≤154 days) (29 408 of 1 278 978 [2.3%]), children who had no link to the father in the Medical Birth Register^14^ or in the Danish Civil Registration System^15^ (13 521 of 1 278 978 [1.1%]), and children whose mothers had filled prescriptions for valproate from 30 days before the first day of the last menstrual period (LMP) and birth (696 of 1 278 978 [0.05%]), leaving 1 235 353 children for analyses. The study was approved by the Danish Data Protection Agency, and all data were analyzed at secured servers at Statistics Denmark using encrypted identification numbers with no contact with the study individuals. The analyses of pseudoanonymous data in Denmark do not require ethical review board approval and do not require participant (or parental) consent according to Danish law. This report followed the Strengthening the Reporting of Observational Studies in Epidemiology (STROBE) reporting guideline for observational studies.

### Medication Exposure During Spermatogenesis

A unique personal identification number is issued by the Danish Civil Registration System^15^ to all individuals born in or immigrating to Denmark. This unique personal identification number ensures complete linkage of individual information across all national registries used in this study. The Danish National Prescription Registery^16^ holds information linked to the personal identification number on all redeemed prescriptions purchased since January 1, 1995 (not including medical treatment given only in hospitals), including information on all prescriptions for ASM filled by patients that are prescribed by hospital physicians, private specialists, or general practitioners.

Paternal valproate exposure was defined as fathers who filled 1 or more prescriptions for valproate from 120 days prior to the beginning of pregnancy (defined as the date of the first day of the mother’s LMP) to conception (LMP plus 14 days) (ie, during the time of spermatogenesis in 3 months prior to conception plus 30 days to account for prescriptions filled just prior to spermatogenesis). Prescriptions for valproate were identified using the Anatomical Therapeutic Chemical (ATC) classification code (N03AG01). In the main analysis, we did not differentiate between valproate being used as monotherapy or valproate being used in combination with other ASMs during spermatogenesis. We estimated the mean daily dose of valproate from the total amount of valproate filled during the exposure period divided by the number of days in the same period and dichotomized the estimated valproate dose into high dose (>750 mg/d) and low dose (≤750 mg/d). For comparison, in one of the sensitivity analyses, we also identified fathers who filled 1 or more prescriptions for lamotrigine (ATC code N03AX09) during the time of spermatogenesis.

### Identification of Paternal and Maternal Epilepsy

The Danish National Patient Registery holds information on admissions to hospitals from 1977 and additionally on inpatient and emergency department visits from 1995.^17,18^ Diagnostic information is recorded using the *International Classification of Diseases, Eighth Revision* (*ICD-8* [until 1993]) and the *International Statistical Classification of Diseases and Related Health Problems, Tenth Revision* (*ICD-10* [from 1994])^19^ for coding. From this register,^17,18^ we identified fathers and mothers with epilepsy diagnosed before LMP minus 120 days (*ICD-8* code, 345 [excluding code 345.29] or *ICD-10* code, G40) (eTable 1 in Supplement 1).

### Identification of Paternal and Maternal Psychiatric Disorders

The Danish Psychiatric Central Research Register provided data on psychiatric disorders for the study population, including all psychiatric hospital contacts (inpatient and, from 1995, outpatient and emergency department visits), based on *ICD* classification.^20^ We identified psychiatric disorders among fathers and mothers as having at least 1 diagnosis of a mental disorder (*ICD-8* codes 290-315; *ICD-10* codes F00-F99)^21^ registered prior to LMP minus 120 days. We stratified the psychiatric diagnoses in a hierarchical manner into bipolar disorder (*ICD-8* codes 296.19, 296.39, and 298.19; *ICD-10* codes F30-31), substance abuse (*ICD-8* codes 291.x9, 294.39, 303.x9, 303.20, 303.28, 303.90, 304, and x9; *ICD-10* codes F10-F19), and other (*ICD-8* codes 290-315; *ICD-10* codes F00-F99). We identified paternal and maternal fillings of prescriptions of psychotropic medication prior to LMP minus 120 days (ATC codes N05A, N05B [excluding code N05BA09], N06A, N06BA01, N06BA02, N06BA04, N06BA09, N06BA11, and N06BA12).

### Information on Congenital Malformations in the Child

We identified major congenital malformations during the child’s first year of life using the primary and secondary diagnoses from the Danish National Patient Register^17,18^ based on *ICD-10* codes^19^ and using the underlying cause of death from the Danish Register of Causes of Death.^22^ We used a Danish adaptation^23^ of the European Network of Population-Based Registries for Epidemiological Surveillance of Congenital Anomalies (EUROCAT) to identify and classify major congenital malformations and specific malformations with high prevalence^24^ (eTable 2 in Supplement 1).

### Information on Neurodevelopmental Disorders in the Child

From the Danish Psychiatric Central Research Register,^21^ we identified children with a diagnosis of neurodevelopmental disorders: intellectual disability (*ICD-10* codes F70-79), disorders of psychological development (*ICD-10* codes F80-83), autism spectrum disorder (*ICD-10* code F84 [excluding codes F84.2-F84.4]), and attention-deficit/hyperactivity disorder (ADHD; *ICD-10* codes F90.0 and F98.8). In further analyses, we specifically considered the subgroup of children with a diagnosis of autism spectrum disorders, and children with neurodevelopmental disorders after excluding diagnoses with disorders of psychological development (eTable 3 in Supplement 1).

### Statistical Analysis

Statistical analysis was performed March 2024. We used log-binomial regression to estimate relative risks (RRs) of congenital malformations, and we estimated HRs with 95% CIs of neurodevelopmental disorders using Cox proportional hazards regression where children were followed up from 1 year of age until death, emigration, a first diagnosis of a neurodevelopmental disorder, or the end of follow-up (December 31, 2018). We adjusted the estimates for the sex of the child, year of birth, and paternal and maternal characteristics, including psychiatric disease, epilepsy diagnosis, age, and highest completed education in the year prior to LMP minus 120 days.^25^ We estimated the cumulative incidence of neurodevelopmental disorders among children exposed or unexposed to paternal valproate use during spermatogenesis. In sibling analyses, we identified siblings with the same father (paternal sibling sets) and used conditional logistic regression to estimate the odds ratio of congenital malformations and stratified Cox proportional hazards regression to estimate HRs of neurodevelopmental disorders.

To assess the robustness of our findings, we compared the risk among valproate-exposed children with several different reference populations: (1) valproate-exposed children compared with unexposed children (main analysis), including dose-response analyses; (2) valproate-exposed children compared with their unexposed paternal siblings (sibling analysis); (3) valproate-exposed children born of fathers with epilepsy compared with valproate-unexposed children born of fathers with epilepsy (all epilepsies and in epilepsy of unknown cause; restriction analysis); (4) valproate-exposed children compared with lamotrigine-exposed children (including an analysis in which valproate-exposed children were matched 1:1 with lamotrigine-exposed children by birth year to account for time trends; active comparator analysis); and (5) valproate-exposed children compared with children born of fathers who filled prescriptions for valproate up to 2 years prior to the exposure period but not during the exposure period (analysis with negative exposure control—meaning an exposure that cannot cause the outcome of interest but shares the same biasing structure that may have been present in the original association).^26^

In sensitivity analyses, we excluded children whose father (LMP minus 120 days to LMP plus 14 days) or mother (LMP minus 30 days to birth) had filled prescriptions for drugs with teratogenic potential^27^ (eTable 4 in Supplement 1). SAS, version 9.4 (SAS Institute Inc) was used to perform the analysis. All *P* values were from 2-sided tests, and results were deemed statistically significant at *P* < .05. There was no adjustment of the significance threshold to account for multiple comparisons, and the analyses should therefore be interpreted as exploratory.

## Results

Among 1 235 353 live births (634 415 boys [51.4%] and 600 938 girls [48.6%]), we identified 1336 children (0.1%) whose fathers had filled prescriptions for valproate during spermatogenesis. The median follow-up was 10.1 years (IQR, 5.1-14.8 years) for valproate-exposed children and 10.3 years (IQR, 5.2-15.6 years) for valproate-unexposed children. The characteristics of children born exposed or unexposed to paternal use of valproate during spermatogenesis are shown in Table 1. Of the 1336 fathers who filled prescriptions for valproate during spermatogenesis, 1052 (78.7%) also received a diagnosis of epilepsy prior to LMP minus 120 days. The median number of filled valproate prescriptions during spermatogenesis was 2 (IQR, 1-3). The number of children with paternal valproate and lamotrigine exposure by birth year is shown in eFigures 1 and 2 in Supplement 1.

**Table 1.** Characteristics of Children With or Without Exposure to Paternal Use of Valproate During Spermatogenesis

Characteristic	Children, No. (%)	
	No paternal valproate use (n = 1 234 017)	Paternal valproate use (n = 1336)
Sex		
Female	600 290 (48.7)	648 (48.5)
Male	633 727 (51.4)	688 (51.5)
Birth year		
1997-2003	427 318 (34.6)	442 (33.1)
2004-2010	421 260 (34.1)	492 (36.8)
2011-2017	385 439 (31.2)	402 (30.1)
Maternal age, y^a^		
<20	17 073 (1.4)	23 (1.7)
20-24	143 236 (11.6)	186 (13.9)
25-29	416 402 (33.7)	437 (32.7)
30-34	436 253 (35.4)	445 (33.3)
35-39	187 858 (15.2)	210 (15.7)
≥40	33 195 (2.7)	35 (2.6)
Paternal age, y^a^		
<20	5096 (0.4)	7 (0.5)
20-24	71 408 (5.8)	93 (7.0)
25-29	300 661 (24.4)	322 (24.1)
30-34	445 844 (36.1)	483 (36.2)
35-39	272 558 (22.1)	280 (21.0)
≥40	138 218 (11.2)	151 (11.3)
Missing	232 (0.02)	0
Maternal educational level^b^		
Primary education or missing	297 390 (24.1)	383 (28.7)
High school or vocational education	492 348 (39.9)	549 (41.1)
Short-cycle higher education	321 713 (26.1)	299 (22.4)
Master’s degree or PhD	122 566 (9.9)	105 (7.9)
Paternal educational level^b^		
Primary education or missing	301 078 (24.4)	447 (33.5)
High school or vocational education	566 111 (45.9)	577 (43.2)
Short-cycle higher education	231 103 (18.7)	208 (15.6)
Master’s degree or PhD	135 725 (11.0)	104 (7.8)
Maternal psychiatric diagnosis^c^		
None	1 149 247 (93.1)	1182 (88.5)
Other	77 553 (6.3)	143 (10.6)
Substance abuse	5653 (0.5)	11 (0.8)
Bipolar disorder	1564 (0.1)	NA^d^
Paternal psychiatric diagnosis^c^		
None	1 181 227 (95.7)	1141 (85.4)
Other	39 753 (3.2)	102 (7.6)
Substance abuse	11 860 (1.0)	47 (3.5)
Bipolar disorder	1177 (0.1)	46 (3.4)
Maternal use of psychotropic medication^e^		
No	1 047 108 (84.9)	1084 (81.1)
Yes	186 909 (15.2)	252 (18.9)
Paternal use of psychotropic medication^e^		
No	1 108 667 (89.8)	958 (71.7)
Yes	125 350 (10.2)	378 (28.3)
Maternal epilepsy diagnosis^f^		
No	1 219 801 (98.9)	1301 (97.4)
Yes	14 216 (1.2)	35 (2.6)
Paternal epilepsy diagnosis^f^		
No	1 222 709 (99.1)	284 (21.3)
Yes	11 308 (0.9)	1052 (78.7)

Abbreviations: LMP, first day of last menstrual period; NA, not applicable.

^a^ At time of childbirth.

^b^ Highest educational level in the year of LMP minus 120 days.

^c^ Psychiatric diagnosis given up until LMP minus 120 days (bipolar disorders: *International Statistical Classification of Diseases and Related Health Problems, Tenth Revision* (*ICD-10*) codes F30-31, *International Classification of Diseases, Eighth Revision* (*ICD-8*) codes 296.19, 296.39, and 298.19; mental and behavioral disorders due to psychoactive substance abuse: *ICD-10* codes F10-F19; *ICD-8* codes 291.x9, 294.39, 303.x9, 303.20, 303.28, 303.90, 304, and x9 [and none of the prior]; other psychiatric disorders: *ICD-10* codes F00-F99, *ICD-8* codes 290-315 [and none of the prior]; no: none of the prior).

^d^ Fewer than 5 observations, here added to the group “Other.”

^e^ Psychotropic medication redeemed up to LMP minus 120 days (Anatomical Therapeutic Chemical codes N05A, N05B [excluding code N05BA09], N06A, N06BA01, N06BA02, N06BA04, N06BA09, N06BA11, and N06BA12).

^f^ Epilepsy diagnosis given up until LMP minus 120 days (*ICD-10* code G40; *ICD-8* codes 345.X [excluding code 345.29]).

### Risk of Major Congenital Malformations

There were 43 903 children (3.6%) who received a diagnosis of major congenital malformations in the first year of life among the 1 235 353 children born in Denmark 1997 to 2017 (eFigure 3 in Supplement 1). When comparing the risk of congenital malformations among valproate-exposed children with that among unexposed children, the adjusted relative risk (ARR) was 0.89 (95% CI, 0.67-1.18) (Table 2). None of the analyses addressing the robustness of this finding (ie, dose-response analyses, sibling analyses, restriction analysis, active comparator analysis, or analysis with negative exposure control) identified increased risk of congenital malformations associated with paternal exposure to valproate during spermatogenesis. Furthermore, restricting the analyses to valproate monotherapy exposure (n = 1017; ARR, 0.83 [95% CI, 0.59-1.17]) and excluding 15 041 children whose parents filled prescriptions for drugs with teratogenic potential (ARR, 0.84 [95% CI, 0.62-1.15]) did not change the results.

**Table 2.** Risk of Congenital Malformations During the First Year of Life Among Children Born of Fathers Who Used Valproate During Spermatogenesis

Comparison	Valproate-exposed children	Reference children	Malformations		Risk estimate (95% CI)	
			Valproate-exposed children	Reference children	Unadjusted	Adjusted^a^
Main analysis^b^	1336	1 234 017	48	43 855	RR, 1.01 (0.77-1.33)	RR, 0.89 (0.67-1.18)
Valproate dose response^c^						
High dose of valproate	715	1 234 017	26	43 855	RR, 1.02 (0.70-1.49)	RR, 0.91 (0.62-1.33)
Low dose of valproate	621		22		RR, 1.00 (0.66-1.50)	RR, 0.87 (0.57-1.32)
Sibling analyses^d^	303	381	10	16	OR, 0.75 (0.32-1.78)	OR, 0.72 (0.30-1.70)
Restriction analysis 1^e^	1052	11 308	35	481	RR, 0.78 (0.56-1.10)	RR, 0.81 (0.58-1.14)
Restriction analysis 2^f^	828	7860	27	342	RR, 0.75 (0.51-1.10)	RR, 0.78 (0.53-1.14)
Active comparator analysis^g^^,^^h^	1336	1663	48	84	RR, 0.71 (0.50-1.01)	RR, 0.67 (0.46-0.98)
Active comparator analysis, matched^i^	1043	1043	38	55	RR, 0.69 (0.46-1.04)	RR, 0.64 (0.42-0.97)
Analysis with negative exposure control^j^	1336	690	48	34	RR, 0.73 (0.47-1.12)	RR, 0.87 (0.54-1.40)

Abbreviations: LMP, first day of last menstrual period; OR, odds ratio; RR, relative risk.

^a^ Adjusted for sex of the child, year of birth, paternal and maternal age at the time of the child’s birth, and paternal and maternal psychiatric diagnosis, psychotropic medication use, epilepsy diagnosis, and highest completed educational level at the time of LMP minus 120 days (sibling analysis is adjusted for sex of the child, year of birth, and paternal and maternal age at the time of the child’s birth).

^b^ Valproate-exposed children compared with unexposed children.

^c^ Children exposed to a high dose and low dose of valproate during spermatogenesis compared with unexposed children.

^d^ Valproate-exposed children compared with unexposed paternal siblings (264 exposure-discordant sibling sets).

^e^ Valproate-exposed children of fathers with epilepsy compared with unexposed children of fathers with epilepsy.

^f^ Valproate-exposed children of fathers with epilepsy of unknown cause compared with unexposed children of fathers with epilepsy of unknown cause.

^g^ Valproate-exposed children compared with lamotrigine-exposed children.

^h^ There were 160 children whose fathers had filled prescriptions for both valproate and lamotrigine during spermatogenesis. In the analyses, these children were included in the group of children whose fathers had filled prescriptions for valproate during spermatogenesis.

^i^ Valproate-exposed children compared with lamotrigine-exposed children, equal number of exposed and unexposed per birth year.

^j^ Valproate-exposed children compared with children of fathers who filled prescriptions for valproate 2 years prior to the exposure period, but not during the exposure period.

We also assessed the risk of specific congenital malformations, but due to the low number of exposed cases, it was possible to provide estimates only for malformations of the cardiac septa. For this specific malformation, there was no increased risk (ARR, 0.53 [95% CI, 0.23-1.19]).

### Risk of Neurodevelopmental Disorders

There were 51 633 children (4.2%) who received a diagnosisis of neurodevelopmental disorders during the study period (eFigure 4 in Supplement 1). The cumulative incidences of neurodevelopmental disorders among children exposed or unexposed to paternal valproate use during spermatogenesis are shown in the Figure. When comparing the risk of neurodevelopmental disorders among valproate-exposed children with that of unexposed children after adjustment for potential confounders, the AHR was 1.10 (95% CI, 0.88-1.37) (Table 3). None of the analyses addressing the robustness of this finding (ie, dose-response analyses, sibling analysis, restriction analysis, active comparator analysis, or analysis with negative exposure control) identified increased risk of neurodevelopmental disorders associated with paternal exposure to valproate during spermatogenesis. When excluding disorders of psychological development from the outcome definition, the results remained largely unchanged (eTable 5 in Supplement 1). Furthermore, restricting the analyses to valproate monotherapy exposure (n = 1017; AHR, 1.13 [95% CI, 0.88-1.44]) and excluding 15 041 children whose parents filled prescriptions for drugs with teratogenic potential (AHR, 1.06 [95% CI, 0.83-1.34]) did not change the results.

**Figure.**
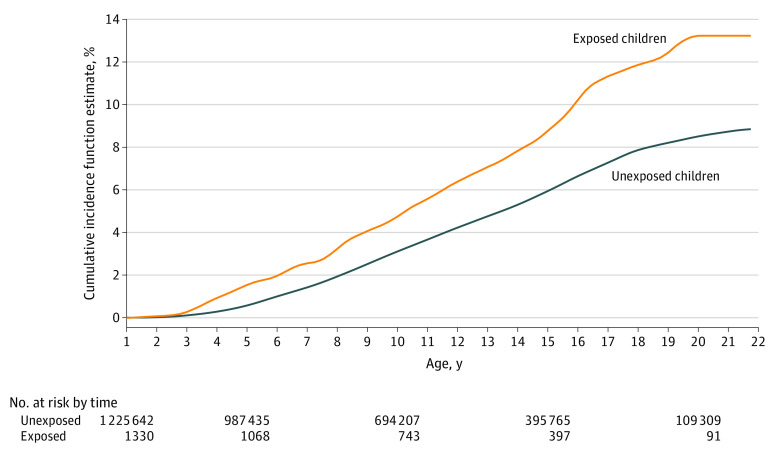
Cumulative Incidence of Neurodevelopmental Disorders Among Children Exposed or Unexposed to Paternal Valproate Use During Spermatogenesis

**Table 3.** Risk of Neurodevelopmental Disorders Among Children Born of Fathers Who Used Valproate During Spermatogenesis

Comparison	Valproate-exposed children^a^	Reference children^a^	Neurodevelopmental disorders		Follow-up time, median (IQR), y		HR (95% CI)	
			Valproate-exposed children	Reference children	Valproate-exposed children	Reference children	Unadjusted	Adjusted^b^
Main analysis^c^	1336	1 234 017	85	51 437	10.1 (5.1-14.8)	10.3 (5.2-15.6)	1.59 (1.28-1.96)	1.10 (0.88-1.37)
Valproate dose response^d^								
High dose of valproate	715	1 234 017	44	51 437	10.5 (5.6-15.2)	10.3 (5.2-15.6)	1.48 (1.10-1.98)	1.10 (0.81-1.49)
Low dose of valproate	621		41		9.7 (4.6-14.5)		1.70 (1.25-2.31)	1.10 (0.80-1.50)
Sibling analyses^e^	303	381	16	23	10.0 (4.9-14.4)	10.4 (6.4-15.3)	1.01 (0.42-2.42)	1.30 (0.51-3.31)
Restriction analysis 1^f^	1052	11 308	70	622	10.4 (5.8-14.7)	8.3 (4.1-13.3)	1.00 (0.78-1.28)	1.09 (0.85-1.39)
Restriction analysis 2^g^	828	7860	62	466	10.9 (6.6-15.2)	9.0 (4.5-13.9)	1.06 (0.81-1.38)	1.13 (0.86-1.47)
Active comparator analysis^h^^,^^i^	1336	1663	85	66	10.1 (5.1-14.8)	6.0 (2.9-10.0)	0.95 (0.68-1.31)	0.97 (0.68-1.38)
Active comparator analysis, matched^j^	1043	1043	56	49	8.2 (4.2-12.2)	8.4 (4.2-12.2)	1.15 (0.79-1.69)	1.03 (0.68-1.57)
Analysis with negative exposure control^k^	1336	690	85	46	10.1 (5.1-14.8)	8.8 (4.3-14.4)	0.87 (0.61-1.25)	0.96 (0.64-1.44)

Abbreviations: HR, hazard ratio; LMP, first day of last menstrual period.

^a^ Shows the number for the whole cohort; 6 exposed and 8375 unexposed died, emigrated, got the outcome, or reached the end of follow-up before age 1 year (start of follow-up) and are not included in these analyses.

^b^ Adjusted for sex of the child, year of birth, paternal and maternal age at the time of the child’s birth, and paternal and maternal psychiatric diagnosis, psychotropic medication use, epilepsy diagnosis, and highest completed educational level at the time of LMP minus 120 days (sibling analysis is adjusted for sex of the child, year of birth, and paternal and maternal age at the time of the child’s birth).

^c^ Valproate-exposed children compared with unexposed children.

^d^ Children exposed to a high dose and low dose of valproate during spermatogenesis compared with unexposed children.

^e^ Valproate-exposed children compared with unexposed paternal siblings (264 exposure-discordant sibling sets).

^f^ Valproate-exposed children of fathers with epilepsy compared with unexposed children of fathers with epilepsy.

^g^ Valproate-exposed children of fathers with epilepsy of unknown cause compared with unexposed children of fathers with epilepsy of unknown cause.

^h^ Valproate-exposed children compared with lamotrigine-exposed children.

^i^ There were 160 children whose fathers had filled prescriptions for both valproate and lamotrigine during spermatogenesis. In the analyses, these children were included in the group of children whose fathers had filled prescriptions for valproate during spermatogenesis.

^j^ Valproate-exposed children compared with lamotrigine-exposed children, equal number of exposed and unexposed children per birth year.

^k^ Valproate-exposed children compared with children of fathers who filled prescriptions for valproate 2 years prior to the exposure period, but not during the exposure period.

### Risk of Autism Spectrum Disorder

There were 24 540 children (2.0%) who received a diagnosis of autism spectrum disorder during the study period (eFigure 5 in Supplement 1). When comparing the risk of autism spectrum disorders among valproate-exposed children with that among unexposed children, the AHR was 0.92 (95% CI, 0.65-1.30) (Table 4). Again, none of the analyses addressing the robustness of this finding identified increased risk of autism associated with paternal exposure to valproate during spermatogenesis. Furthermore, restricting the analyses to valproate monotherapy exposure (n = 1017; AHR, 0.82 [95% CI, 0.54-1.24]) and excluding 15 041 children whose parents filled prescriptions for drugs with teratogenic potential (AHR, 0.91 [95% CI, 0.63-1.32]) did not change the results.

**Table 4.** Risk of Autism Spectrum Disorders Among Children Born of Fathers Who Used Valproate During Spermatogenesis

Comparison	Valproate-exposed children^a^	Reference children^a^	Autism spectrum disorder		Follow-up time, median (IQR), y		HR (95% CI)	
			Valproate-exposed children	Reference children	Valproate-exposed children	Reference children	Unadjusted	Adjusted^b^
Main analysis^c^	1336	1 234 017	34	24 479	10.5 (5.5-15.2)	10.5 (5.3-15.7)	1.31 (0.93-1.83)	0.92 (0.65-1.30)
Valproate dose response^d^								
High dose of valproate	715	1 234 017	13	24 479	10.6 (5.9-15.6)	10.5 (5.3-15.7)	0.90 (0.52-1.55)	0.66 (0.38-1.15)
Low dose of valproate	621		21		10.1 (4.9-14.6)		1.81 (1.18-2.77)	1.20 (0.78-1.86)
Sibling analyses^e^	303	381	7	17	10.5 (5.1-14.5)	10.4 (6.5-15.4)	0.39 (0.10-1.47)	0.54 (0.14-2.12)
Restriction analysis 1^f^	1052	11 308	27	298	10.7 (6.0-15.1)	8.5 (4.1-13.5)	0.80 (0.54-1.19)	0.85 (0.57-1.26)
Restriction analysis 2^g^	828	7860	27^h^	219	11.4 (6.9-15.6)	9.3 (4.5-14.2)	0.83 (0.54-1.28)	0.87 (0.56-1.33)
Active comparator analysis 1^i^^,^^j^	1336	1663	34	43	10.5 (5.5-15.2)	6.1 (2.9-10.1)	0.56 (0.36-0.89)	0.68 (0.41-1.13)
Active comparator analysis 2^k^	1043	1043	21	31	8.4 (4.3-12.4)	8.5 (4.2-12.4)	0.68 (0.39-1.18)	0.76 (0.42-1.38)
Analysis with negative exposure control^l^	1336	690	34	21	10.5 (5.5-15.2)	9.1 (4.4-14.8)	0.77 (0.45-1.33)	0.83 (0.45-1.54)

Abbreviation: HR, hazard ratio.

^a^ Shows the number for the whole cohort; 6 exposed and 8291 unexposed died, emigrated, got the outcome, or reached the end of follow-up before age 1 year (start of follow-up) and are not included in these analyses.

^b^ Adjusted for sex of the child, year of birth, paternal and maternal age at the time of the child’s birth, and paternal and maternal psychiatric diagnosis, psychotropic medication use, epilepsy diagnosis, and highest completed educational level at the time of the child’s birth (sibling analysis is adjusted for sex of the child, year of birth, and paternal and maternal age at the time of the child’s birth).

^c^ Valproate-exposed children compared with unexposed children.

^d^ Children exposed to a high dose and low dose of valproate during spermatogenesis compared with unexposed children.

^e^ Valproate-exposed children compared with unexposed paternal siblings (264 exposure-discordant sibling sets).

^f^ Valproate-exposed children of fathers with epilepsy compared with unexposed children of fathers with epilepsy.

^g^ Valproate-exposed children of fathers with epilepsy of unknown cause compared with unexposed children of fathers with epilepsy of unknown cause.

^h^ This number is valproate-exposed children of fathers with epilepsy, as the difference to valproate-exposed children of fathers with epilepsy of unknown cause is small (<5) and therefore cannot be shown.

^i^ Valproate-exposed children compared with lamotrigine-exposed children.

^j^ There were 160 children whose fathers had filled prescriptions for both valproate and lamotrigine during spermatogenesis. In the analyses, these children were included in the group of children whose fathers had filled prescriptions for valproate during spermatogenesis.

^k^ Valproate-exposed children compared with lamotrigine-exposed children, equal number of exposed and unexposed per birth year.

^l^ Valproate-exposed children compared with children of fathers who filled prescriptions for valproate 2 years prior to the exposure period, but not during the exposure period.

## Discussion

In this study of more than 1 million births identified in Danish health care registers, we were unable to identify an increased risk of congenital malformations and neurodevelopmental disorders among children who were born to fathers who filled prescriptions for valproate during spermatogenesis. The findings were robust and persisted when we compared the risk among children with paternal valproate exposure with the risk in the overall population, when assessing the risk in dose response and sibling analyses, when restricting the analyses to fathers with epilepsy and epilepsy with unknown underlying cause, when comparing with the children of fathers with lamotrigine exposure, when taking time trends into account, and when comparing with children born of fathers who discontinued valproate use. Thus, we were unable to replicate the findings recently reported by PRAC.^11,12,13^

Danish health registers have previously been able to identify adverse outcomes associated with maternal exposure to ASMs (including valproate),^28,29,30^ and we should therefore expect that we would be able to identify similar adverse outcomes associated with paternal exposure, should they exist. It is therefore puzzling that we were unable to identify the risk of neurodevelopmental disorders reported by PRAC, especially because our analyses are based partly on the same register data.^11,12,13^ However, our findings are in accordance with a previous study using Swedish register data that found no association of paternal ASM use during conception with congenital malformations and neurodevelopmental disorders among the offspring.^31^ This Swedish study found that epilepsy among fathers (regardless of ASM use during spermatogenesis) was associated with a slight increased risk of neurodevelopmental outcomes among offspring, supporting careful consideration when choosing comparison groups in observational studies.^31^ Another study from Norway in 2013 was based on self-reported paternal epilepsy and ASM use among 653 fathers in the 6 months leading up to conception.^32^ In this study, abnormal scores for personal-social skills and autistic traits at 18 months of age were more common after paternal use of ASM leading up to conception, but this association was not found at 36 months of age. This study did not report on offspring risk after paternal use of specific ASMs, including valproate, leading up to conception.

There could be methodological differences that may explain the differences between our results and those reported by PRAC.^11,12,13^ Several factors need to be considered when using large health care databases for drug safety surveillance in association with pregnancy, including follow-up, cohort definition, and study design.^33^

### Limitations

This study has some limitations. Use of ASM has changed over time,^23^ and the incidence of neurodevelopmental disorders (eg, autism spectrum disorder)^34^ has increased in the study period, as also found in this study. Although we tried to take these time trends into account by matching the exposure group by birth year and by adjusting estimates for year of birth and follow-up, a potential difference in accounting for time trends between our study and the study reported by PRAC may explain the differences. On the other hand, the proportion of children with a diagnosis of major congenital malformations varied only slightly over time, suggesting that, for this outcome, the time trends are less likely to have skewed comparisons between exposed and unexposed children. Like the study reported by PRAC,^13^ we were unable to identify an increased risk of congenital malformations among children who were exposed to valproate during spermatogenesis. We assessed several specific congenital malformations but were able to provide risk estimates only for malformations of the cardiac septa; for this outcome, the risk was not increased. In sensitivity analyses, excluding children whose parents filled prescriptions for drugs with teratogenic potential did not change any of the findings.

The association between maternal use of valproate during pregnancy and risk of congenital malformations has been shown in several studies,^6,7^ but it remains unknown whether the underlying pathology of this risk of congenital malformations differs from the underlying pathology of the risk of neurodevelopmental disorders identified after maternal use of valproate during pregnancy.^8,9^ Similarly, we do not know whether any risk of neurodevelopmental disorders associated with valproate exposure during spermatogenesis would share pathologic pathways with a risk of congenital malformations. In our study, we included only live births, and the study does not include terminations of pregnancy and particularly did not include terminations of pregnancy for fetal anomaly, as there was no paternal link for these pregnancies. Consequently, major congenital malformations, which are mainly diagnosed prenatally, may not be very well captured in the present study, resulting in a potential underestimation of the overall valproate-related risk of congenital malformations.^23,35,36^

We found no increased risk in dose-response analyses and when restricting analyses to children exposed to valproate monotherapy during spermatogenesis. In the analyses restricted to children born of fathers with epilepsy, the crude HR of developmental disorders decreased compared with the main analyses, suggesting that paternal underlying disorders may contribute to a risk of neurodevelopmental disorders in the child. In active comparator analyses, we identified children whose fathers had filled prescriptions for lamotrigine during spermatogenesis (ie, similar to what had been done in the PRAC study).^13^ Although maternal lamotrigine exposure during pregnancy is not associated with increased risk of congenital malformations in the offspring,^6^ the lower risk of malformations in children whose fathers filled prescriptions for valproate compared with lamotrigine during spermatogenesis suggests that this group of children may not constitute an optimal comparison group for exposure during spermatogenesis.

Parental epilepsy was identified from hospital, outpatient, and emergency department diagnoses. Thus, we may not have captured patients seen exclusively for epilepsy by their private practitioner or private neurologist. However, we identified patients seen in hospitals, outpatient departments, and emergency departments from 1977 onward, including admission for other conditions in which epilepsy was recorded as a supplementary diagnosis. Among children with paternal exposure to valproate during spermatogenesis, 78.7% were born to fathers who also received a diagnosis of epilepsy prior to spermatogenesis, suggesting that we would capture most fathers with epilepsy, as valproate is also used for other indications (eg, bipolar disorder).

Our definition of neurodevelopmental disorders includes intellectual disability, disorders of psychological development, autism spectrum disorders, and ADHD. Although the definition of neurodevelopmental disorders is not clearly stated in the PRAC study report,^13^ we believe that these disorders are most often considered neurodevelopmental.^20^ The validity of neurodevelopmental disorder diagnoses as comprehensively used in this study has not been estimated, but a validation study of the autism spectrum disorder diagnosis reported a positive predictive value of 94%.^37^ We addressed the importance to the outcome definition in 2 sensitivity analyses (ie, in analysis restricting the outcome to children with autism spectrum disorder and another analysis excluding diagnoses of disorders of psychological development). None of these analyses suggested that valproate exposure during spermatogenesis was associated with adverse neurodevelopmental outcomes.

Men with epilepsy may face a difficult decision when deciding on the best use of ASM for seizure prevention because valproate has consistently been shown to be the best-tolerated drug for generalized epilepsy,^4,5^ and emerging human evidence of the potential adverse effects associated with valproate use in the context of reproduction stems mainly from observational studies. Observational studies (including ours) may be subject to varying degrees of confounding,^33^ and, accordingly, we adjusted for several potential confounders, including parental education and comorbid psychiatric disorders. Like the study reported by PRAC,^13^ our study had an active comparator (exposure to lamotrigine during spermatogenesis). Lamotrigine is usually considered a safe alternative for women of fertile age with epilepsy when considering maternal use of ASM during pregnancy and was therefore viewed as a reasonable comparator for the exposure during spermatogenesis in the PRAC study. However, the use of lamotrigine has increased considerably over the study period, meaning that the follow-up time in children with paternal lamotrigine exposure is shorter than in children with paternal valproate exposure, which is associated with the probability of having detected a neurodevelopmental disorder. On the other hand, over time, diagnostic rates of many neurodevelopmental disorders, such as autism and ADHD, have increased dramatically,^20,34^ and the probability of receiving a diagnosis of neurodevelopmental disorders is also highly age specific, suggesting a complex interplay among age, cohort, and calendar period effects, which makes any direct comparisons difficult. These issues may introduce bias when using lamotrigine as a comparator for valproate, where the exposure during spermatogenesis did not show the same time trend. To circumvent this problem, we carried out a separate analysis, where valproate-exposed children were matched 1:1 to lamotrigine-exposed children by birth year, thereby ensuring that the 2 cohorts of children experienced similar age, cohort, and calendar period effects. Results from this analysis still showed no increased risk of neurodevelopmental disorders nor of congenital malformations among children with paternal valproate exposure, suggesting that the lack of signal could not be explained by these time trends.

Among the variables used for adjustment, there was missing information on paternal age for 232 of 1 235 353 fathers (0.02%). Parental education was missing for less than 5% of children and was categorized together with “primary education.” Given the low proportion of missing data, we believe that this does not contribute to significant bias in the analyses. Furthermore, we did not have information on smoking status of fathers, which is another limitation of the study. We included paternal and maternal diagnoses of substance abuse prior to spermatogenesis as separate variables. Substance abuse is a relevant confounder for the risk of both congenital malformations and neurodevelopmental disorders but may not be fully captured by the hospital diagnoses, and residual unmeasured confounding may thus still be an issue. We used paternal and maternal use of psychotropic medication prior to spermatogenesis as indicators of psychiatric disorders in the parents and thus potential confounders for neurodevelopmental outcomes. However, we did not capture medically untreated psychiatric disorders, which constitutes another limitation of the study. There was limited information on methods in the PRAC study on how confounding was handled^13^; however, it is revealed that the history of paternal and maternal neurodevelopmental disorders was used as an exclusion criterion in the PRAC study. Misclassification of parental neurodevelopmental disorders (ie, parents not being diagnosed, despite being affected) is, however, likely to be substantial, and such misclassification is expectedly more pronounced in the earlier study period (when valproate use was more common) than in the later period (when lamotrigine use was more common), due to the increasing diagnostic trends of neurodevelopmental disorders; this differential misclassification could potentially introduce a bias.

## Conclusions

In all analyses based on this large Danish cohort study, exposure to valproate during spermatogenesis was not associated with risk of congenital malformations or neurodevelopmental disorders, including autism, among offspring. Our findings do not support the results in the study reported by the European Medicines Agency PRAC and warrant further studies.

## Notes

Supplement 1. **eTable 1.** Diagnostic Classification of Underlying Causes of Epilepsy With Underlying Etiology (“Secondary Epilepsy”) According to International Classification of Disease (ICD) codes) – Version 10 (ICD-10) and Equivalent Version 8 (ICD-8) Diagnoses **eTable 2.** ICD 10 Codes Used for the Identification of Major Congenital Malformations in the First Year of Life **eTable 3.** ICD 10 Codes Used for the Identification of Neurodevelopmental Disorders **eTable 4.** ATC Codes Used for the Identification of Paternal Use of Teratogenic Drugs During Spermatogenesis (LMP – 120 to LMP + 14), and Maternal Use of Teratogenic Drugs During Pregnancy (LMP – 30 to Birth) **eTable 5.** Risk of Neurodevelopmental Disorders Excluding Disorders of Psychological Development in Children Born of Fathers Who Used Valproate During Spermatogenesis **eFigure 1.** Number of Children Born to Fathers Using Valproate During Spermatogenesis by Birth Year (Children Born in Denmark, 1997 to 2017) **eFigure 2.** Number of Children Born to Fathers Using Lamotrigine During Spermatogenesis by Birth Year (Children Born in Denmark, 1997 to 2017) **eFigure 3.** Number of Children Identified With Major Congenital Malformations During the First Year of Life by Birth Year (Children Born in Denmark, 1997 – 2017) **eFigure 4.** Cumulative Incidence of Neurodevelopmental Disorders During Follow-Up by Birth Year (Children Born in Denmark, 1997 – 2017) **eFigure 5.** Cumulative Incidence of Autism Spectrum Disorders During Follow-Up by Birth Year (Children Born in Denmark, 1997 – 2017)

Supplement 2.
Data Sharing Statement
