# Supplementary material for: Valproate Use During Spermatogenesis and Risk to Offspring
Source: JAMA Netw Open. 2024 Jun 4;7(6):e2414709. doi: 10.1001/jamanetworkopen.2024.14709 (PMC11151155; doi:10.1001/jamanetworkopen.2024.14709)
Supplement: Supplement 1. — eTable 1. Diagnostic Classification of Underlying Causes of Epilepsy With Underlying Etiology (“Secondary Epilepsy”) According to International Classification of Disease (ICD) codes) – Version 10 (ICD-10) and Equivalent Version 8 (ICD-8) Diagnoses eTable 2. ICD 10 Codes Used for the Identification of Major Congenital Malformations in the First Year of Life eTable 3. ICD 10 Codes Used for the Identification of Neurodevelopmental Disorders eTable 4. ATC Codes Used for the Identification of Paternal Use of Teratogenic Drugs During Spermatogenesis (LMP – 120 to LMP + 14), and Maternal Use of Teratogenic Drugs During Pregnancy (LMP – 30 to Birth) eTable 5. Risk of Neurodevelopmental Disorders Excluding Disorders of Psychological Development in Children Born of Fathers Who Used Valproate During Spermatogenesis eFigure 1. Number of Children Born to Fathers Using Valproate During Spermatogenesis by Birth Year (Children Born in Denmark, 1997 to 2017) eFigure 2. Number of Children Born to Fathers Using Lamotrigine During Spermatogenesis by Birth Year (Children Born in Denmark, 1997 to 2017) eFigure 3. Number of Children Identified With Major Congenital Malformations During the First Year of Life by Birth Year (Children Born in Denmark, 1997 – 2017) eFigure 4. Cumulative Incidence of Neurodevelopmental Disorders During Follow-Up by Birth Year (Children Born in Denmark, 1997 – 2017) eFigure 5. Cumulative Incidence of Autism Spectrum Disorders During Follow-Up by Birth Year (Children Born in Denmark, 1997 – 2017) [file jamanetwopen-e2414709-s001.pdf]

Supplementary Online Content

Christensen J, Trabjerg BB, Dreier JW. Valproate use during spermatogenesis and risk to offspring. *JAMA Netw Open.* 2024;7(6):e2414709.
doi:10.1001/jamanetworkopen.2024.14709

eTable 1. Diagnostic Classification of Underlying Causes of Epilepsy With Underlying Etiology (“Secondary Epilepsy”) According to International Classification of Disease (ICD) codes) – Version 10 (ICD-10) and Equivalent Version 8 (ICD-8) Diagnoses
eTable 2. ICD 10 Codes Used for the Identification of Major Congenital Malformations in the First Year of Life
eTable 3. ICD 10 Codes Used for the Identification of Neurodevelopmental Disorders
eTable 4. ATC Codes Used for the Identification of Paternal Use of Teratogenic Drugs During Spermatogenesis (LMP – 120 to LMP + 14), and Maternal Use of Teratogenic Drugs During Pregnancy (LMP – 30 to Birth)
eTable 5. Risk of Neurodevelopmental Disorders Excluding Disorders of Psychological Development in Children Born of Fathers Who Used Valproate During Spermatogenesis
eFigure 1. Number of Children Born to Fathers Using Valproate During Spermatogenesis by Birth Year (Children Born in Denmark, 1997 to 2017)
eFigure 2. Number of Children Born to Fathers Using Lamotrigine During Spermatogenesis by Birth Year (Children Born in Denmark, 1997 to 2017)
eFigure 3. Number of Children Identified With Major Congenital Malformations During the First Year of Life by Birth Year (Children Born in Denmark, 1997 – 2017)
eFigure 4. Cumulative Incidence of Neurodevelopmental Disorders During Follow-Up by Birth Year (Children Born in Denmark, 1997 – 2017)
eFigure 5. Cumulative Incidence of Autism Spectrum Disorders During Follow-Up by Birth Year (Children Born in Denmark, 1997 – 2017)

This supplementary material has been provided by the authors to give readers additional information about their work.

© 2024 Christensen J et al. *JAMA Network Open.*

eTable 1. Diagnostic Classification of Underlying Causes of Epilepsy With Underlying Etiology ("Secondary Epilepsy") According to International Classification of Disease (ICD) codes) – Version 10 (ICD-10) and Equivalent Version 8 (ICD-8) Diagnoses

**Disease**		**ICD-8 codes**	**ICD-10 codes**
Infections	CNS infections	013, 027.01, 036.09, 040-043, 045- 046, 052.01, 053.02, 054.03, 055.01, 056.01, 062-065, 071.99, 072.02, 075.01, 079.29, 090.49, 094.9, 320, 322-324, 474	A02.2C, A06.6, A17, A22.9C, A32.1, A39.0, A50.4, A51.4B, A52.1A-B, A54.8A, A54.8D, A80-89, B00.3-00.4, B01.0-01.1, B02.0-02.1, B05.0-05.1, B06.0, B26.1-26.2, B37.5, B45.1, B58.2, B60.2, E23.6A, G00-09
	Tetanus	037.99, 670.04	A33-35
	Malaria	084	B50-54
	Cysticercosis	123.19	B69.0
	Cystic echinococcis	122.89, 122.99	B67.3-67.4, B67.8-67.9
Conditions originating in the perinatal period.		760-779	P00-P96
Malformations		740-759	Q00-Q99
Brain tumors		191, 198.39, 198.40, 198.41, 225	C70-72, C79.3 D32-D33
Traumatic brain injury		850.99, 800.99-801.09, 803.99, 851.29-854.99	S06.0, S02.0-S02.1, S02.7, S02.9, S06.1-S06.9
Stroke		430-438	I60-69

We classified fathers' epilepsy into epilepsy with underlying etiology ("secondary epilepsy") if the first epilepsy diagnosis was preceded by any of the following conditions: CNS infections, traumatic brain injury, stroke, brain neoplasm, perinatal complications, or congenital malformations, and epilepsy with unknown underlying etiology ("idiopathic epilepsy") if the first epilepsy diagnosis was not preceded by any of these conditions.

© 2024 Christensen J et al. *JAMA Network Open.*

eTable 2. ICD 10 Codes Used for the Identification of Major Congenital Malformations in the First Year of Life

Malformation group	Included codes (incl subgroups)	Excluded minor malformations
All major malformations	Q00-99, D181A, D215, D821, P350, P351, P371	Q078D, Q078G, Q101-Q105, Q135, Q170-Q175, Q179-Q182, Q184-Q187, Q189, Q211C, Q246, Q254E, Q261, Q314, Q318H, Q320, Q322, Q331, Q357, Q381, Q382, Q385B, Q400, Q401, Q430, Q444, Q458B, Q501, Q502, Q505, Q523, Q525, Q527, Q53, Q544, Q552F, Q552B, Q610, Q627, Q633, Q653-Q656, Q658, Q659, Q661- Q669, Q670-Q675, Q678, Q680, Q682A, Q683, Q684, Q685, Q740G, Q752, Q753, Q760, Q764L, Q765, Q766A-Q766C, Q767C, Q825, Q833, Q845, Q846, Q899, Q95
**Specific malformations**	**Included codes (incl subgroups)**	
Spina bifida:	Q05	
Malformation of cardiac septa:	Q21	
Cleft lip and cleft palate:	Q35-Q37	
Other congenital malformations of the digestive system:	Q38-Q45	
Hypospadias	Q54	
Malformation of the urinary system:	Q60-Q64	
Polydactyly:	Q69	

© 2024 Christensen J et al. *JAMA Network Open.*

eTable 3. ICD 10 Codes Used for the Identification of Neurodevelopmental Disorders

	**Included codes (incl subgroups)**
**Neurodevelopmental disorders**	Intellectual disability (ICD-10: F70-79)<br>Disorders of psychological development (ICD 10: F80-83)<br>Autism spectrum disorders (ICD 10: F84 (excl F84.2-F84.4))<br>ADHD (ICD 10: F90.0+F98.8)
**Autism Spectrum Disorder**	F84 (excl F84.2-F84.4)
**Neurodevelopmental disorders, excluding diagnoses with disorders of psychological development**	Intellectual disability (ICD-10: F70-79)<br>Autism spectrum disorders (ICD 10: F84 (excl F84.2-F84.4))<br>ADHD (ICD 10: F90.0+F98.8)

Among the total population of 51,633 children diagnosed with neurodevelopmental disorders, there were 34,474 (66.8%) diagnosed with only one type of neurodevelopmental disorder, 14,569 (28.2%) diagnosed with two types of neurodevelopmental disorders, 2,411 (4.7%) diagnosed with three types of neurodevelopmental disorders and 179 (0.3%) diagnosed with all four types of neurodevelopmental disorders.

© 2024 Christensen J et al. *JAMA Network Open.*

**eTable 4.** ATC Codes Used for the Identification of Paternal Use of Teratogenic Drugs During Spermatogenesis (LMP – 120 to LMP + 14), and Maternal Use of Teratogenic Drugs During Pregnancy (LMP – 30 to Birth)
Retinoids: D10AD, D05BB, D10BA, L01XF<br><br>Angiotensin-converting enzyme inhibitors: C09A, C09B<br><br>Vitamin K antagonists: B01AA<br><br>Lithium: N05AN01<br><br>Carbamazepine: N03AF01<br><br>Oxcarbazepine: N03AF02<br><br>Phenytoin: N03AB02<br><br>Phenobarbital: N03AA02<br><br>Methotrexate: L04AX03 L01BA01

LMP: First day of the last menstrual period.

© 2024 Christensen J et al. *JAMA Network Open.*

eTable 5. Risk of Neurodevelopmental Disorders Excluding Disorders of Psychological Development in Children Born of Fathers Who Used Valproate During Spermatogenesis

Comparison	Valproate exposed children[b]	Reference children[b]	Neurodevelopmental disorder cases[a]		Follow-up time, median (IQR), y		HR estimate (95% CI)	
			Valproate Exposed	Reference children	Valproate Exposed	Reference children	Unadjusted	Adjusted[c]
**1a. Main analysis[d]**	1 336	1 234 017	76	47 671	10.2 (5.2 - 14.8)	10.4 (5.2 - 15.6)	1.53 (1.22 - 1.91)	1.06 (0.84 - 1.34)
**1b. Valproate dose-response[e]**								
**High dose of valproate**	715	1 234 017	44[f]	47 671	10.5 (5.7 - 15.2)	10.4 (5.2 - 15.6)	1.37 (1.00 - 1.89)	1.03 (0.74 - 1.42)
**Low dose of valproate**	621		41[f]		9.8 (4.7 - 14.5)		1.70 (1.24 - 2.34)	1.10 (0.80 - 1.52)
**2. Sibling analyses[g]**	303	381	16[f]	23[f]	10.0 (4.9 - 14.4)	10.4 (6.4 - 15.4)	0.96 (0.39 - 2.39)	1.27 (0.48 - 3.38)
**3a. Restriction analysis[h,i]**	1 052	11 308	63	576	10.4 (5.9 - 14.7)	8.4 (4.1 - 13.4)	0.97 (0.75 - 1.26)	1.06 (0.82 - 1.38)

© 2024 Christensen J et al. *JAMA Network Open*.

**3b. Restriction analysis – fathers with epilepsy of unknown etiology[j]**	828	7 860	56	432	11.0 (6.7 - 15.2)	9.0 (4.5 - 14.0)	1.03 (0.78 - 1.37)	1.10 (0.84 - 1.46)
**4a. Active comparator analysis[k]**	1 336	1 663	76	66 [f]	10.2 (5.2 - 14.8)	6.0 (2.9 - 10.1)	0.85 (0.61 - 1.19)	0.87 (0.60 - 1.26)
**4b. Active comparator analysis – equal number of exposed and unexposed per year[l]**	1 043	1 043	50	49 [f]	8.3 (4.3 - 12.2)	8.4 (4.2 - 12.2)	1.05 (0.71 - 1.56)	0.95 (0.62 - 1.46)
**5. Analysis with negative exposure control[m]**	1 336	690	76	40	10.2 (5.2 - 14.8)	8.9 (4.4 - 14.5)	0.90 (0.61 - 1.32)	1.07 (0.69 - 1.65)

Abbreviations: HR, hazard ratio; CI, confidence interval; IQR, interquartile range; LMP, first day of last menstrual period.

[a] Excluding disorders of psychological development from the definition of neurodevelopmental disorders

[b] Shows N for the whole cohort 6 exposed and 8,353 unexposed died, emigrated, got the outcome or reached end of follow-up before age 1 years (start of follow-up) and are not included in these analyses.

[c] Adjusted for sex of the child, year of birth, paternal and maternal age at the time of the child's birth, and paternal and maternal psychiatric diagnosis, psychotropic medication use, epilepsy diagnosis, and highest completed educational level at the time of LMP minus 120 days (sibling analysis is adjusted for sex of the child, year of birth, and paternal and maternal age at the time of the child's birth).

[d] Valproate-exposed children compared with unexposed children.

[e] Children exposed to a high dose and low dose of valproate during spermatogenesis compared with unexposed children.

[f] This number is the numbers for neurodevelopmental disorders overall from the mail analysis as the difference to neurodevelopmental disorders excluding disorders of psychological development is small (<5) and can therefore not be shown.

[g] Valproate-exposed children compared with unexposed paternal siblings (264 exposure-discordant sibling sets).

© 2024 Christensen J et al. *JAMA Network Open.*

[h] Valproate-exposed children of fathers with epilepsy compared with unexposed children of fathers with epilepsy.
[i] Valproate-exposed children of fathers with epilepsy of unknown cause compared with unexposed children of fathers with epilepsy of unknown cause.
[j] Valproate-exposed children compared with lamotrigine-exposed children.
[k] There were 160 children whose fathers had filled prescriptions for both valproate and lamotrigine during spermatogenesis. In the analyses, these children were included in the group of children whose fathers had filled prescriptions for valproate during spermatogenesis.
[l] Valproate-exposed children compared with lamotrigine-exposed children, equal number of exposed and unexposed per birth year.
[m] Valproate-exposed children compared with children of fathers who filled prescriptions for valproate 2 years prior to the exposure period, but not during the exposure period.

© 2024 Christensen J et al. *JAMA Network Open.*

eFigure 1. Number of Children Born to Fathers Using Valproate During Spermatogenesis by Birth Year (Children Born in Denmark, 1997 to 2017)

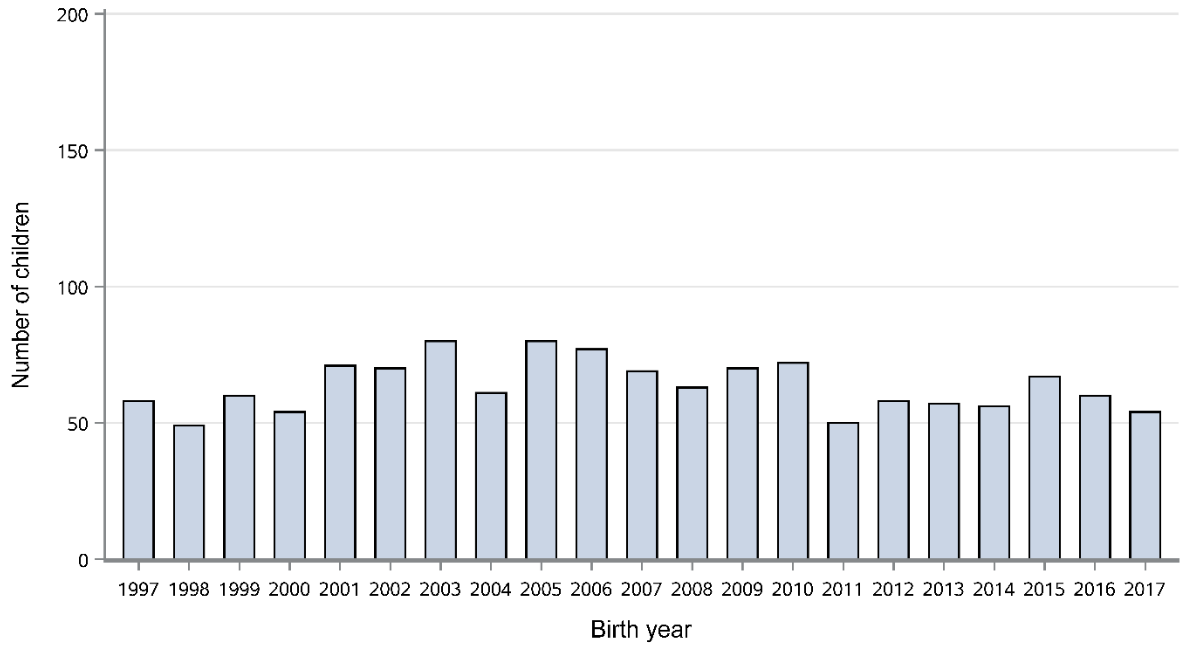

Overall, we identified 1,336 (0.1%) children where the father had filled prescriptions for valproate during spermatogenesis.

© 2024 Christensen J et al. *JAMA Network Open.*

eFigure 2. Number of Children Born to Fathers Using Lamotrigine During Spermatogenesis by Birth Year (Children Born in Denmark, 1997 to 2017)

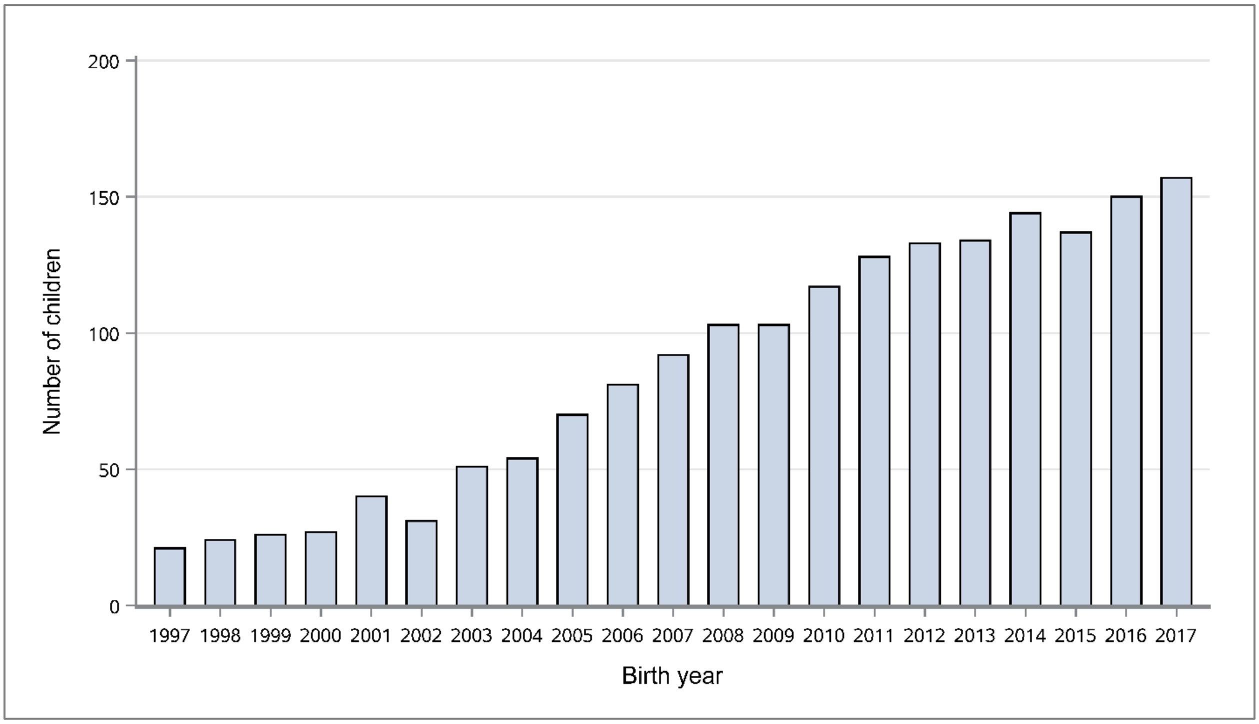

Overall, we identified 1,823 (0.2%) children where the father had filled prescriptions for lamotrigine during spermatogenesis.

© 2024 Christensen J et al. *JAMA Network Open.*

eFigure 3. Number of Children Identified With Major Congenital Malformations During the First Year of Life by Birth Year (Children Born in Denmark, 1997 – 2017)

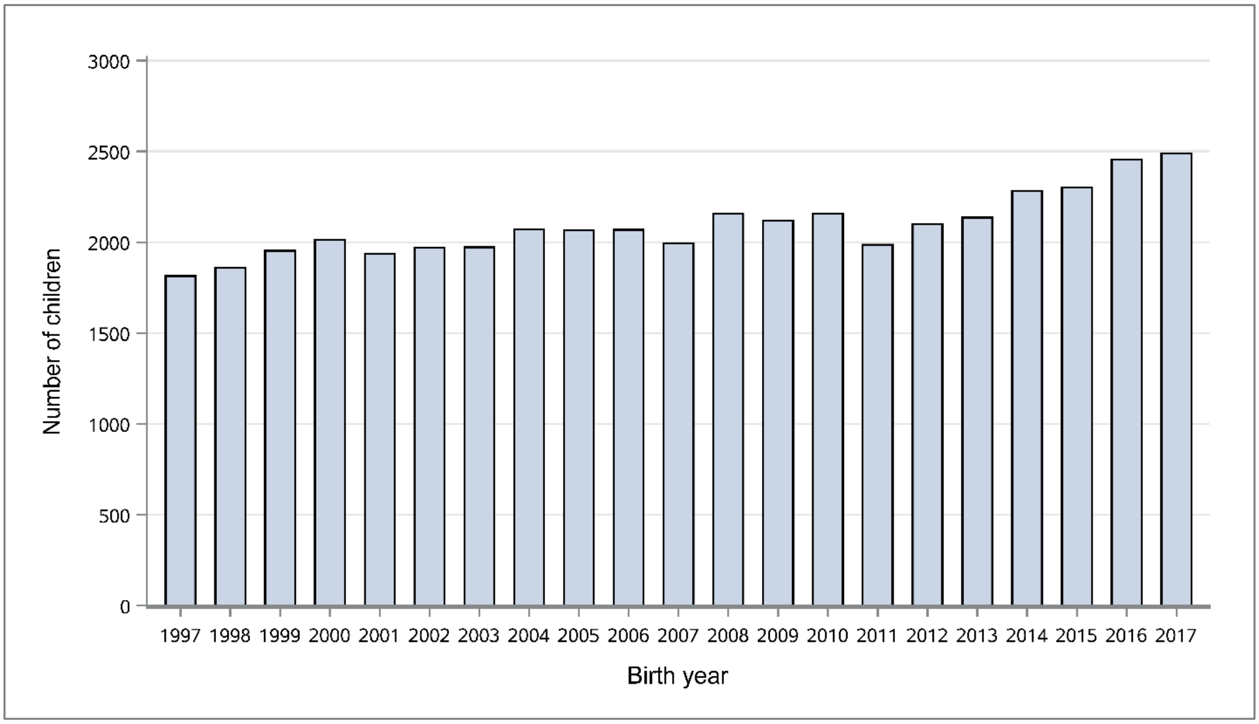

Overall, we identified 43,903 (3.6%) children with major congenital malformations during the first year of life.

© 2024 Christensen J et al. *JAMA Network Open.*

eFigure 4. Cumulative Incidence of Neurodevelopmental Disorders During Follow-Up by Birth Year (Children Born in Denmark, 1997 – 2017)

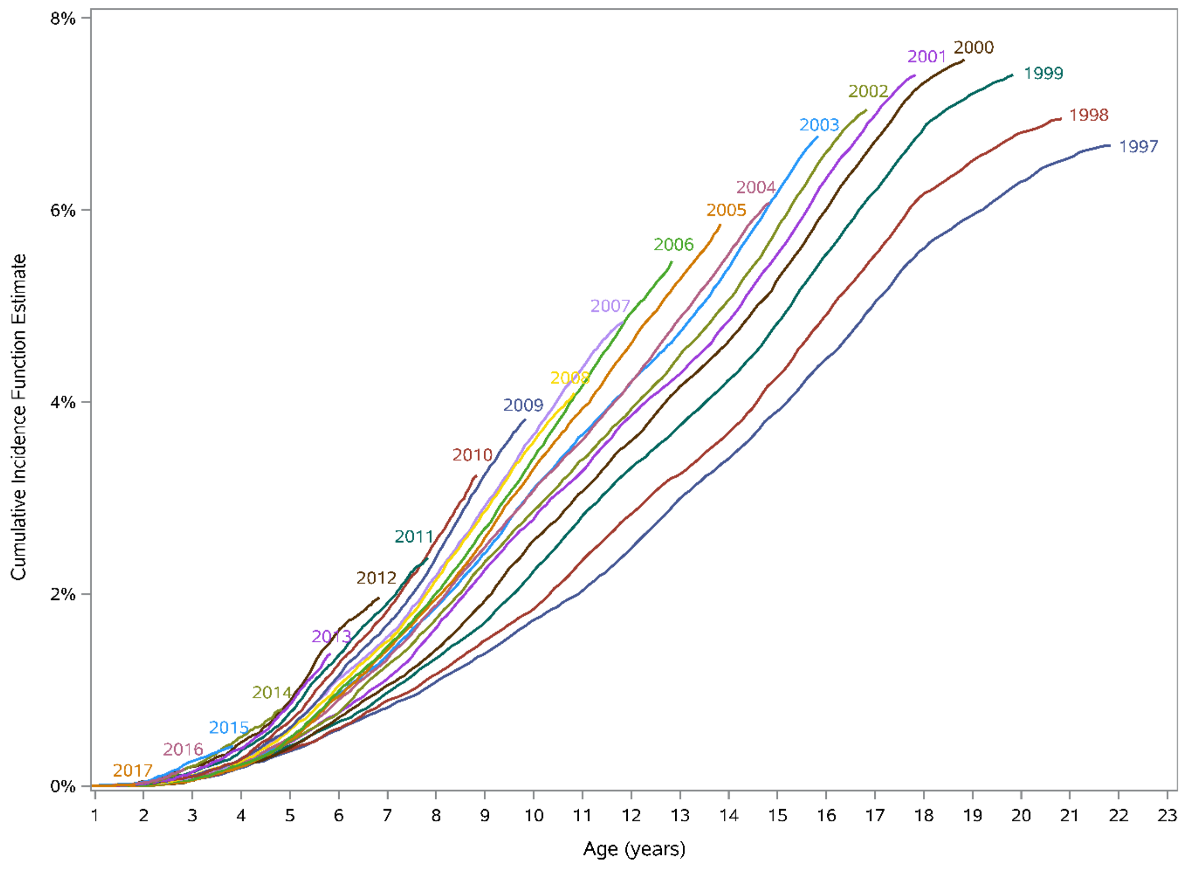

There were 51,633 (4.2%) children diagnosed with neurodevelopmental disorders during follow up among 1,235,353 children born in Denmark 1997 to 2017

© 2024 Christensen J et al. *JAMA Network Open.*

eFigure 5. Cumulative Incidence of Autism Spectrum Disorders During Follow-Up by Birth Year (Children Born in Denmark, 1997 – 2017)

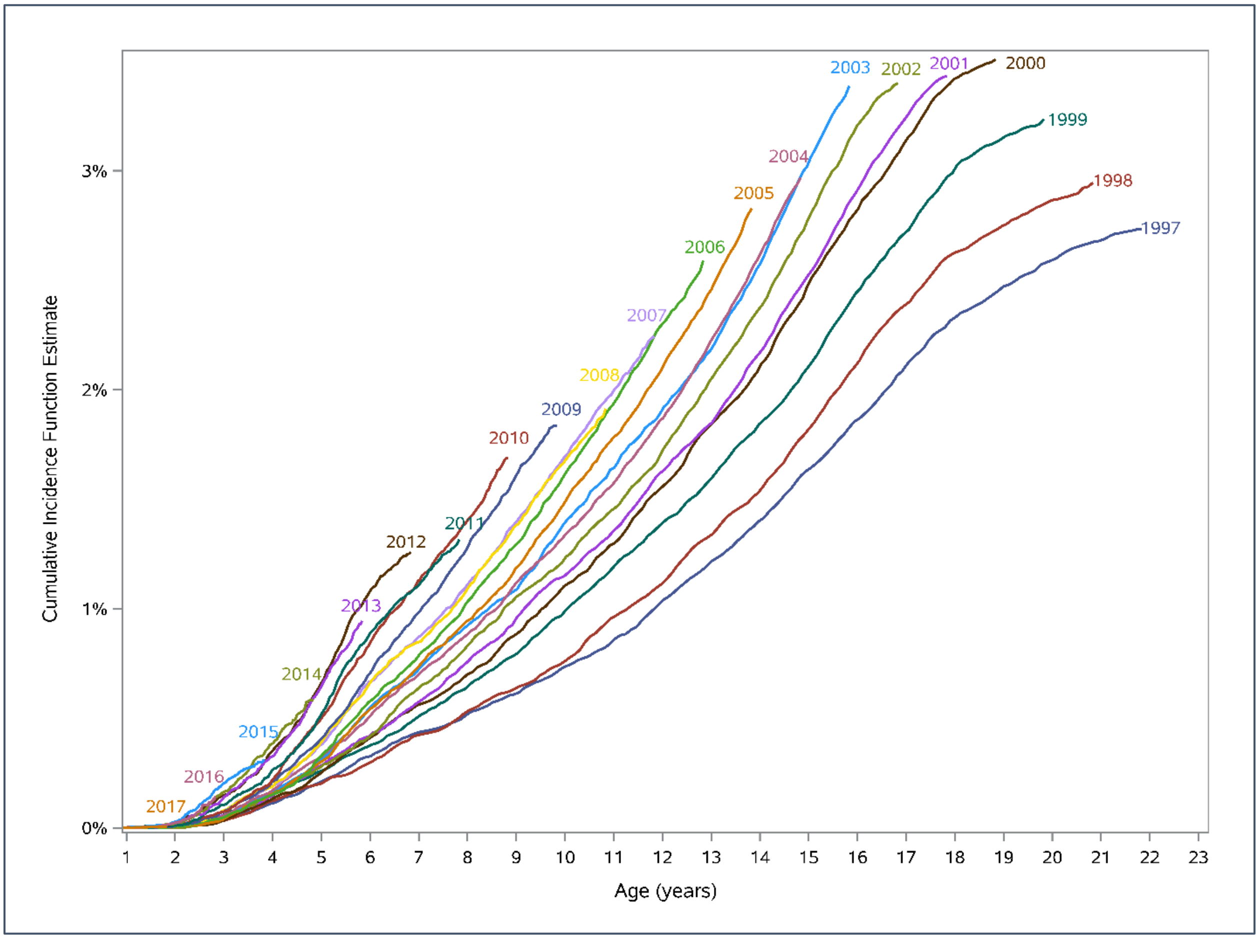

There were 24,540 (2.0%) children diagnosed with autism spectrum disorder during follow up among 1,235,353 children born in Denmark 1997 to 2017

© 2024 Christensen J et al. *JAMA Network Open.*
