# Supplementary material for: Valproate Use During Spermatogenesis and Risk to Offspring
Source: JAMA Netw Open. 2024 Jun 4;7(6):e2414709. doi: 10.1001/jamanetworkopen.2024.14709 (PMC11151155; doi:10.1001/jamanetworkopen.2024.14709)
Supplement: Supplement 2. — Data Sharing Statement [file jamanetwopen-e2414709-s002.pdf]

Data Sharing Statement

Christensen. Valproate Use During Spermatogenesis and Risk to Offspring. *JAMA Netw Open*. Published June 04, 2024. doi:10.1001/jamanetworkopen.2024.14709

Data
Data available: No

Additional Information
Explanation for why data not available: Data were based on Danish national registers, and individual level data cannot be shared. However, summary statistics, in addition to the results provided in the results section and supplementary material, may be provided on request.
